# Risk perception and the influence on uptake and use of biomedical prevention interventions for HIV in sub-Saharan Africa: A systematic literature review

**DOI:** 10.1371/journal.pone.0198680

**Published:** 2018-06-14

**Authors:** Emily A. Warren, Pauline Paterson, William S. Schulz, Shelley Lees, Robyn Eakle, Jonathan Stadler, Heidi J. Larson

**Affiliations:** 1 Department of Public Health, Environments, and Society, London School of Hygiene & Tropical Medicine, London, United Kingdom; 2 Department of Infectious Disease Epidemiology, London School of Hygiene & Tropical Medicine, London, United Kingdom; 3 Department of Global Health and Development, London School of Hygiene & Tropical Medicine, London, United Kingdom; 4 Wits Reproductive Health and HIV Institute, Johannesburg, South Africa; 5 Department of Anthropology and Development Studies, University of Johannesburg, Johannesburg, South Africa; 6 Department of Global Health, University of Washington, Seattle, United States of America; Cardiff University, UNITED KINGDOM

## Abstract

**Background:**

Risk perception has been found to be a crucial factor explaining inconsistent or non-use of HIV prevention interventions. Considerations of risk need to expand beyond risk of infection to also include the personal, social, emotional, and economic risks associated with prevention intervention use.

**Objectives:**

This systematic review of qualitative peer-reviewed literature from sub-Saharan Africa examines perceptions of risk associated with HIV infection and HIV prevention intervention use.

**Data sources:**

We searched Medline, Embase, PsychInfo, Africa Wide Info, CINAHL, and Global Health for publications and screened them for relevance.

**Study eligibility criteria:**

Peer-reviewed qualitative studies published since 2003 were eligible for inclusion if they examined risk perception or uncertainty in the context of a medically regulated intervention. Only studies focusing on adults were included.

**Study appraisal and synthesis methods:**

Included publications were quality assessed using the Hawker method and coded thematically.

**Results:**

10318 unique papers were identified, of which 29 are included. Among the themes identified, a particularly salient one was the potential of HIV prevention interventions to threaten the stability of a relationship and impact on how and when people may—or may not—choose to use prevention interventions.

**Limitations:**

This literature review excludes grey-literature, which may have distinct valuable insights. We also excluded quantitative studies that may have challenged or triangulated our findings.

**Conclusions and implications:**

When considering the risk of HIV acquisition, it is insufficient to examine biological risk in isolation from the personal, relational and economic costs associated with intervention use. This loss of emotional, physical, or material support may be perceived as more consequential than the prevention of a potential infection.

## Introduction

The development and testing of new HIV prevention interventions aims to prevent new HIV infections as well as provide both women and men with a greater range of intervention choices. While several new modalities are promising, effectiveness in preventing HIV requires good adherence. Research has identified risk perception as a crucial variable in explaining non-use of HIV prevention interventions, showing that when situations and relationships are perceived as being low-risk, it inhibits motivations to use and adhere to prevention interventions.[1–3]

The basic concept of risk, as the probability that an event will happen,[4] is fundamental for epidemiologists, researchers, and health workers. However, people are not only at risk of a disease, but also consequences of interventions themselves including physical, social, and psychological risks associated with their use. These may include consequences as significant as stigma, or as personal as the termination of a relationship.[5] Recognizing this complexity, there has been a move away from examining decisions about HIV risk and intervention use as a purely rational cost-benefit analysis, towards an approach that includes a contextualization of people’s subjectivities and experiences as central to their motivation to use or not use interventions.[6] This recognition is important for two key reasons: firstly, it acknowledges that decisions occur in a context that can both restrain and enable choice,[7] and secondly, HIV falls within a much larger and complex hierarchy of concerns extending beyond a disease and entering into deeply personal realms of trust, love, economic security, and values.[6] This constructivist approach to understanding risk highlights that an “individual’s biographic characteristics,”[6] one’s relationship with partner(s), social network norms, and context all influence how people perceive and respond to the risk of HIV. This broader conception of risk is useful because, unlike in biomedical frameworks, health is not assumed to be the key factor motivating decision-making.[6]

Throughout the development of HIV prevention interventions from condoms, pre and post exposure prophylaxes (PrEP and PEP), voluntary medical male circumcision (VMMC), to emerging interventions, including microbicides, vaginal rings, and injectable ARVs, concerns have been raised about risk compensation, or the increase in risky behaviours caused by a decrease in either real or perceived disease risk.[8]

The question guiding this research is: How do individuals understand the risks associated with HIV and HIV prevention, and how do these conceptualisations influence the use of prevention interventions? We examine the notion of risk perception across HIV prevention interventions, both in development and currently available, to compare and contrast how risk is perceived and influences decision-making. While HIV-testing is not always considered a prevention intervention, it is included in this review because it is a well-known intervention and knowing one’s HIV status was considered an important factor influencing the use of other interventions.[9]

## Methods

### Search strategy

We focused the review on evidence from sub-Saharan Africa due to the high-burden of HIV/AIDS, and availability of qualitative research, so that there was some general comparability of context. The search strategy was built through an iterative process of developing concepts based on the research aim. After defining concepts, a search was conducted for related search strategies and literature reviews to ensure comprehensiveness of search terms and synonyms.[10] Because the primary concept, risk, does not translate directly into many languages spoken in sub-Saharan Africa, background research was conducted on other terms that have been used to study similar concepts. After consultations with other researchers who have conducted similar systematic literature reviews and a trained information scientist with a background in HIV systematic reviews, the search strategy was further refined to include terms relating to: HIV prevention *and* risk perception *and* service uptake *and* qualitative research *and* sub-Saharan Africa.

For the detailed search strategy, see S1 Table.

We searched the bibliographic databases Africa Wide Info, CINAHL, Embase, Global Health, Medline, and PsychInfo. We exported all citations into Endnote (version 7) and removed duplicates. Two authors screened the papers for relevance by title and abstract. Any paper that at least one author thought was relevant was brought forward into the full-text screening. Additional papers were identified through reference chasing, whereby reference lists of potentially relevant publications were screened. Lists of papers for inclusion and exclusion were compared and any discrepancies were discussed amongst authors until resolved. The original search was run 6 March 2014 and updated using an identical search strategy on 3 March 2016. The update was felt necessary after a break in the analysis as many on the study team started working on the Ebola outbreak in West Africa.

### Inclusion criteria

**Fig 1:**
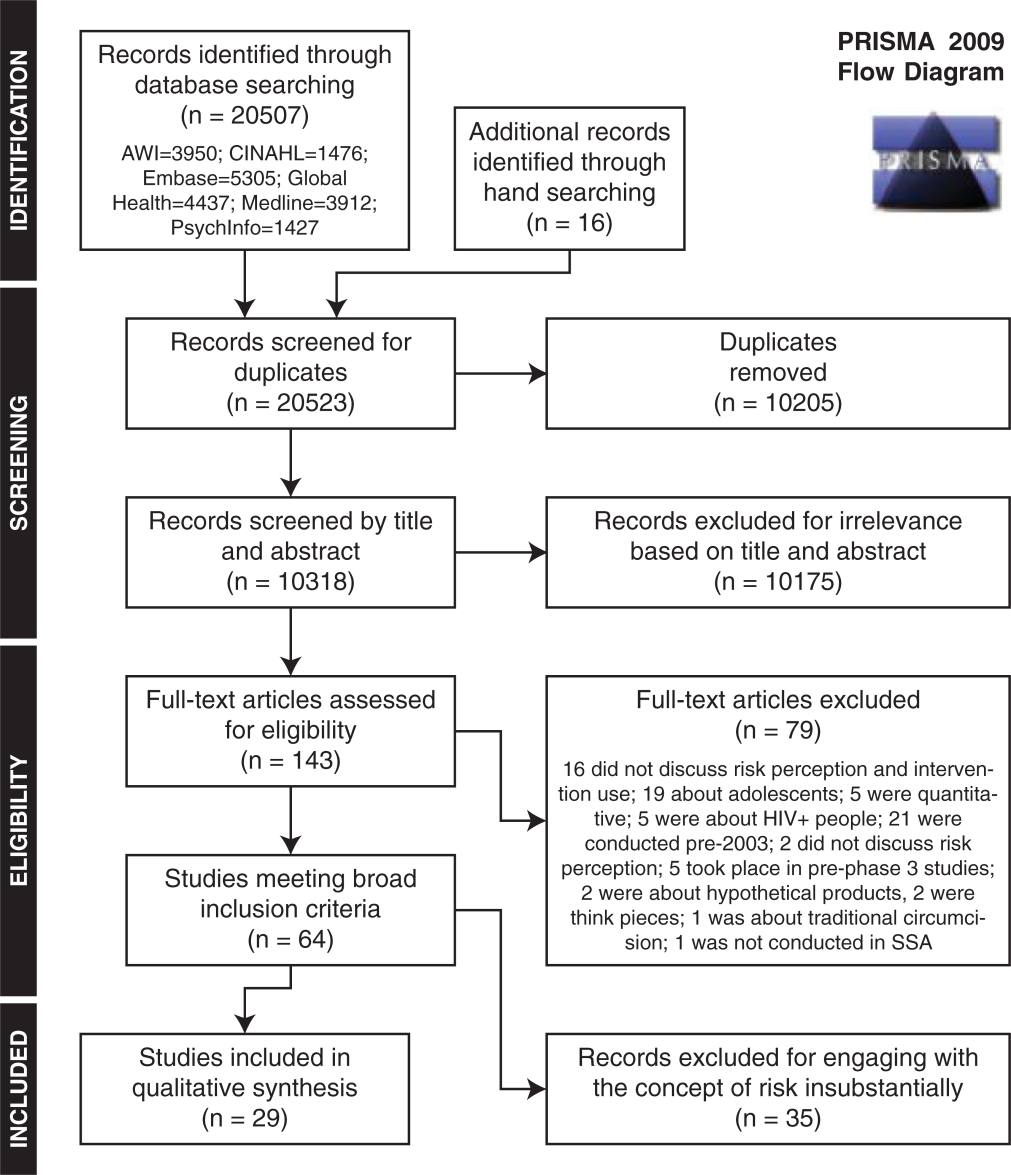
PRISMA flow diagram.

To be included, publications needed to be qualitative, peer-reviewed research conducted with adults in sub-Saharan Africa. Papers were included if they examined risk perception or uncertainty in the context of a medically regulated HIV prevention intervention. This included interventions such as condoms, microbicides, HIV testing, PrEP, PEP, and VMMC but excluded economic or structural interventions. Papers focusing on young people under 18-years old were excluded as their challenges around HIV prevention and intervention use may be distinct from those of adults.[11] Papers published before 2003 were also not eligible for inclusion. Quantitative studies were also excluded because of the better epistemological appropriateness for answering this question using qualitative studies. Data were extracted from papers written in English, Spanish, or French (Fig 1).

### Quality assessment

The quality of each included study was assessed using the Hawker et al framework which accounts for methodological appropriateness and reporting.[12] In it, there are nine domains: abstract and title; introduction and aims; methods and data; sampling; data analysis; ethics and bias; findings/results; transferability/generalizability; implications and usefulness. Each domain is ranked 1 for ‘very poor’ to 4 for ‘good.’ Scores therefore rank between 9 and 36. Studies were deemed high-quality (A) 30–36 points; medium quality (B) 24–29 points; low quality (C), 9–23 points.[13] Studies were not excluded for being poor quality. Weaknesses were also accounted for by recording author and reviewer identified limitations in the extraction table, which was used when synthesising and interpreting the findings.

### Analysis and data synthesis

Using Microsoft Excel, data, including study location and design, methods used, population, and sample size were extracted from the papers. EW read the papers and created a list of risks associated with HIV, interventions explored, and contextual factors that were reported as influencing either risk perception or intervention use. These factors were organized thematically and simplified into a proposed coding framework. Each of the other authors then coded five randomly selected papers and suggested modifications to the coding framework. Where needed, definitions of codes were created and refined by the study team. The coding framework and the full text of the included papers were loaded into QSR NVivo 10 (qualitative data analysis software) for final coding. All studies were coded in duplicate by two authors.

Since the codes were intentionally broad, the text included within each code was separated and organised thematically. Each theme was then summarised and analysed in relation to the research question. When similar themes emerged under different codes, they were merged in order to improve clarity and depth of understanding. The headings generated from the qualitative narrative synthesis were used to organize the most salient themes. The papers from the original and updated search were treated identically. For clarity and concision, the results have been combined and are presented in the same PRISMA flowchart.

Sixty-four papers were found to be relevant to our research question. In order to focus the review on papers that addressed the issue of risk perception and intervention use most directly, the papers were classified on a scale of 1–4. This four-point scale was used because the appropriate cut-off point for inclusion was not obvious at the outset, and so rather than risk needing to revise the criteria and have all coders re-classify the papers, we adopted a graded classification scale, allowing us to decide the appropriate cut-off point later. Papers were coded as “1” if risk perception and intervention use were part of their primary research question. Papers were coded as “2” if risk perception and intervention use was a major theme, but not their primary research question. Papers were coded as “3” if they discussed risk perception explicitly, with intervention use as an implicitly related issue, or vice-versa. Papers coded as “4” mentioned the connection between risk perception and intervention use only peripherally. Papers were coded in duplicate and discrepancies were discussed between authors until consensus was reached. Only papers coded as 1 and 2 are discussed in detail here as those classified as “3” or “4” contributed very little substance to the qualitative synthesis.[14–49] Thus, of the 64 papers that met the general inclusion criteria (See Fig 1), only the 28 papers coded as “1” or “2” have been included in the detailed analysis presented below.

## Results

### Description of studies

Seven studies were conducted in South Africa,[50–56] four each in Kenya[50, 57–59] and Malawi.[60–63] Three studies each were conducted in Mozambique[64–66] and Nigeria.[67–69] Two studies each were conducted in Uganda,[70, 71] Zambia,[72, 73] and Zimbabwe.[51, 74] One study each was conducted in Ghana,[75] Madagascar,[76] Swaziland,[77] and Tanzania,[78] Some studies were conducted in more than one country. Only one study focused on men who have sex with men (MSM).[58] Condoms were the most commonly researched intervention. A description of the included studies is included in Table 1. A matrix showing the distribution of research by populations and interventions is shown in Table 2.

**Table 1:** Description of included studies.

Author	Title	Year	Country	Method^1^	Intervention	Quality Assessment^2^
Abbott et al.[73]	Female sex workers, male circumcision and HIV: a qualitative study of their understanding, experience, and HIV risk in Zambia	2013	Zambia	IDI	Condoms, VMMC	C
Achan et al.[75]	Coping strategies of young mothers at risk of HIV/AIDS in the Kassena-Nankana district of Northern Ghana	2009	Ghana	FGD, IDI	Condoms	C
Ankomah et al.[67]	HIV-related risk perception among female sex workers in Nigeria	2011	Nigeria	FGD	Condoms	C
Bandali[64]	Norms and practices within marriage which shape gender roles, HIV/AIDS risk and risk reduction strategies in Cabo Delgado, Mozambique	2010	Mozambique	FGD, IDI	Condoms	C
Chirwa et al.[60]	HIV prevention awareness and practices among married couples in Malawi	2011	Malawi	IDI	Condoms, HIV Testing	C
Corneli et al.[50]	A descriptive analysis of perceptions of HIV risk and worry about acquiring HIV among FEM-PrEP participants who seroconverted in Bondo, Kenya and Pretoria, South Africa	2014	Kenya South Africa	Mixed-method, SSI	Condoms, HIV testing, PrEP	A
Grund & Hennink[77]	A Qualitative Study of Sexual Behavior Change and Risk Compensation Following Adult Male Circumcision in Urban Swaziland	2001	Swaziland	IDI	VMMC	B
Haram[78]	AIDS and risk: The handling of uncertainty in northern Tanzania	2006	Tanzania	Ethnography	Condoms, HIV Testing	C
Izugbara[68]	Constituting the unsafe: Nigerian sex workers’ notions of unsafe sexual conduct	2007	Nigeria	FGD, IDI, KII, Observation	Condoms	C
Kacanek et al.[51]	A qualitative study of obstacles to diaphragm and condom use in an HIV prevention trial in sub-Saharan Africa	2012	South Africa Zimbabwe	FGD, IDI	Condoms, Diaphragm	B
Kalipeni & Ghosh[61]	Concern and practice among men about HIV/AIDS in low socioeconomic income areas of Lilongwe, Malawi	2007	Malawi	FGDs, IDI	Condoms	C
Katsinde et al.[74]	Student nurses' perceptions of the HIV and AIDS problem: a case study of Bindura School of Nursing, Zimbabwe	2011	Zimbabwe	Interviews	Condoms, HIV Testing	C
Kumwenda et al.[62]	Factors shaping initial decision-making to self-test amongst cohabiting couples in urban Blantyre, Malawi	2014	Malawi	IDI	HIV self-testing	B
L’Engle et al.[57]	Understanding partial protection and HIV risk and behavior following voluntary medical male circumcision rollout in Kenya	2014	Kenya	IDI	VMMC	B
Langa et al.[65]	HIV risk perception and behavior among sex workers in three major urban centers of Mozambique	2014	Mozambique	FGD, IDI	Condoms, HIV testing and counselling (HTC)	B
Mkandawire et al.[63]	‘At risk by fact of birth’: perceptions and concerns about medical male circumcision for HIV prevention in northern Malawi	2014	Malawi	FGD, IDI	VMMC	C
Muñoz et al.[69]	They bring AIDS to us and say we give it to them': Socio-structural context of female sex workers' vulnerability to HIV infection in Ibadan Nigeria	2010	Nigeria	FGD, IDI, Observation	Condoms, HIV Testing	B
Munyewende et al.[52]	Exploring perceptions of HIV risk and health service access among Zimbabwean migrant women in Johannesburg: A gap in health policy in South Africa	2011	South Africa	IDI	Condoms	C
Ohnishi & Notico[66]	Reduction of health-related risks among female commercial sex workers: Learning from their life and working experiences	2011	Mozambique	FGD, KII	Condoms	C
Okal et al.[58]	Social context, sexual risk perceptions and stigma: HIV vulnerability among male sex workers in Mombasa, Kenya	2009	Kenya	FGD, IDI	Condoms	B
Parker et al.[53]	Concerns about partner infidelity are a barrier to adoption of HIV-prevention strategies among young South African couples	2014	South Africa	Couples IDI	Condoms, HTC	A
Reiss et al.[59]	When I Was Circumcised I Was Taught Certain Things”: Risk Compensation and Protective Sexual Behavior among Circumcised Men in Kisumu, Kenya	2010	Kenya	Interviews	VMMC	B
Sahin-Hodoglugil et al.[56]	Degrees of disclosure: a study of women's covert use of the diaphragm in an HIV prevention trial in sub-Saharan Africa	2009	South Africa Zimbabwe	FGD, IDI	Condom, Diaphragm, Lubricant gel	B
Shefer et al.[55]	AIDS fatigue and university students' talk about HIV risk	2012	South Africa		Condoms	B
Sikasote et al.[72]	Voluntary counselling and testing for HIV in a Zambian mining community: serial interviews with people testing negative	2011	Zambia	FGD, initial and follow-up interviews	VCT	B
Siu et al.[70]	Masculinity, social context and HIV testing: an ethnographic study of men in Busia district, rural eastern Uganda	2014	Uganda	IDI, Participant Observation	HTC	A
Stern et al.[54]	Sexual and reproductive health perceptions and practices as revealed in the sexual history narratives of South African men living in a time of HIV/AIDS	2014	South Africa	FGD, IDI	Condoms, HTC	A
Stoebenau et al.[76]	"…but then he became my Sipa": the implications of relationship fluidity for condom use among women sex workers in Antananarivo, Madagascar	2009	Madagascar	IDI, Interviews, Participant Observation, SSI	Condoms	C
Ware et al.[71]	What's love got to do with it? Explaining adherence to oral antiretroviral pre-exposure prophylaxis for HIV serodiscordant couples	2012	Uganda	IDI	PrEP	B

1-FGD = Focus group discussion; IDI = in-depth interview; KII = key informant interview, SSI-semi-structured interview

2-High quality studies, marked A received 30–36 points; medium quality studies, marked B, received 24–29 points; low quality studies, marked C, received 9–23 points.

**Table 2:** Intervention and population matrix of included studies (n = 28).

	Condoms	Diaphragm/ Microbicide	HIV testing	PrEP	VMMC
Sex workers (M/F)	[73]; [67]; [68];[69]; [66]; [76]; [65]		[69]; [66]; [65]		[73]
General population	[78]; [54]		[78]; [72]; [54]		
Couples (committed/ married/ cohabitating)	[64]; [60]; [53]		[60]; [62]; [53]		
Men	[77]; [61]; [59]		[70]		[77]; [59]; [57]; [63]
Migrants	[52]				
MSM	[58]				
People in sero-discordant partnerships				[71]	
Students	[74]; [55]		[74]		
Trial participants	[50]; [51]; [56]	[51]; [56]		[50]	
Young mothers	[75]				

### Thematic areas

The finding are presented thematically, relating to the role of trust, partner influence, autonomy, and the intervention’s symbolic meaning. While these themes emerged from the data, they sometimes overlap, largely reflecting the complexity of peoples’ lives. These are the multifaceted aspects of risk perception but we are conscious that they are not discrete. Where themes were relevant to both sex-workers and non-sex workers, results are presented together, highlighting similarities. Findings particularly relevant for sex workers are addressed separately at the end of the findings section.

#### Risk perception decreases when feelings of trust grow in relationships

Condoms were reportedly more acceptable in casual relationships not characterized by expectations of commitment or fidelity.[53, 54, 64] As relationships became more committed, continued use was difficult to maintain.[50, 51, 54, 58, 60, 64, 69, 74–76] The trust between sexual partners, generated over time, was displayed through eschewing HIV prevention and was found to influence people to prioritize the relationship and their emotional wellbeing over HIV prevention.[74] In a study on the HIV risk perception of student nurses in Zimbabwe, a male student said, “One may use them [condoms] when you have sexual contact with a lover for the first time, but later on people tend to stop using them because of the trust. Love is about trust and if one continues to insist on the use of condoms, then no love exists.”[74] Likewise, a male informant in Stern et al’s study of sexual history narratives in South Africa said, “when you are with someone you use a condom the first time you have sex with them, second time, by the third time you are used to that person and you almost trust them. No one uses a condom longer than that.”[54] When condom use symbolises mistrust, abandoning them marks the transition from a casual, transactional, or emotionally uninvested relationship to one that is based on love, trust, and commitment.[50–53, 58, 60, 61, 64, 66, 68, 69, 74, 76] Condom use was therefore particularly problematic within marriage, since it was understood to reflect mistrust and suspicions of affairs.[51, 53, 58, 60, 64, 74]

Insisting on condom use was reportedly used to punish one’s partner, or to communicate dissatisfaction or a breakdown of trust. Parker et al researched concerns around fidelity among young South African couples. One woman said, “We use condoms when I’m angry, when I am thinking that he is cheating on me. But apart from that we are not using condoms.”[53] Patterns of condom use demonstrate the fluidity of risk perception even when the risk itself may be stable: women felt physically or materially vulnerable when they could not trust their partner, and less at risk when they were confident in their partner’s fidelity.

Fears that men were not trustworthy frequently emerged as a theme, as did women’s need for HIV prevention interventions they could use without their partner’s knowledge.[51, 53, 56, 73, 74, 78] One woman in Parker’s study said, “You know I don’t want to trust someone anymore and even now I don’t trust my boyfriend. I love him but I don’t trust him 100%.”[53] While this lack of trust was not ideal for either partner, it was not felt to be sufficient grounds for ending a relationship.

Corneli et al (2014) interviewed participants from Kenya and South Africa who seroconverted whilst enrolled in FEM-PrEP, a PrEP efficacy trial in Kenya, South Africa, and Tanzania.[50] Participants expressed deep hurt at becoming HIV positive, largely because they trusted their partners. One woman said, ‘‘I also have one sexual partner, whom I trusted and knew cannot make me get there [be HIV positive].”[50] Some women’s faith in the protective power of trust, the normalization of men’s multiple concurrent partnerships, and few women-controlled HIV prevention interventions places women at a higher risk when trying to balance the desire to feel loved while protecting their health.

#### Partner behaviour influences risk perception

As shown above, one’s HIV risk perception is heavily influenced by their partner’s suspected or actual behaviour, which either made partners feel safer or increasingly vulnerable.

Even when women considered their own behaviour to be low-risk, their partner’s actions left them vulnerable to infection.[50, 64, 75] A woman in Bandali et al’s study in Mozambique stated, “I always explained to him that there are diseases. I can die, he can die and so can these women he is with. When I talked to my husband about it he told me to shut up, that I know nothing and that he is a man and knows everything.”[64] In a PrEP study in Kenya and South Africa, respondents who acknowledged they were at moderate to high risk of HIV infection felt so because of uncertainty of their partner’s monogamy or HIV status.[50] A participant from Bondo, Kenya explained, ‘‘[HIV] is something I knew was there and I could get it at any time … because I know my status but I don’t know his status. I don’t know his sexual behaviour. I just know my sexual behaviour.”[50]

In a study of PrEP adherence, study participants often felt that trying to protect their health was futile without the support, cooperation, or fidelity of their partner.[71] For these women, surrendering to the inevitability of HIV was the most feasible option: “I began to suspect he is seeing other women… is he sleeping with other women so he can acquire more infection and pass it on to me? [This makes me] so angry that I feel it's useless to keep taking this medicine [PrEP]. Because of that, I decided to leave it.”[71]

Suspecting or knowing about partners’ other relationships was a common theme.[50, 53, 60, 62, 64, 71, 72, 75, 78] While men’s concurrent partnerships were seen as more commonplace than women’s, they were also viewed as a source of infection.[50, 53, 60, 62, 64, 71, 72, 75, 78] Young mothers in Ghana and married women in Mozambique encouraged their husbands to use condoms during extramarital affairs to protect themselves from HIV infection.[64, 75]

Men sometimes acknowledged the validity of women’s health concerns relating to their other partners, even while continuing these relationships: “Traditionally a man can marry as many wives as he wants. So, our wives know they cannot win the argument if they complain about our relations with other women. Now that there are diseases especially AIDS… When she complains about the risk of disease, you may try to defend yourself but you know that honestly your wife has a point.”[75]

#### Intervention use is not always an individual’s choice

Regardless of how at-risk someone feels, most interventions, especially those widely available, are difficult to use covertly. As shown above and below, respondents reported male resistance to interventions, limiting their use and pressuring their partners to decide whether to insist on intervention use or risk ending a relationship.

In all of the studies included in this review, men were presented as having greater social power in determining intervention use. It was commonly reported that women would want to use some form of prevention but were discouraged or disallowed. Reports of men resisting or sabotaging condoms[50, 51, 54, 56, 58, 64, 65, 67–69, 71, 73, 75, 76, 78] and refusing HIV testing[60, 62, 70, 74] were common. A woman from a study on young mothers in northern Ghana summarised her frustration: “What can you do to prevent your husband from infecting you with AIDS? Are you going to buy the female condom and insert it while sleeping every night?… It is rather the man who can prevent it but if he doesn’t like using condoms, then there is little you can do to prevent yourself from being infected.”[75] A man in Zimbabwe remarked, “The condom requires a lot of work, especially for me. When I am tired and in the mood to have sex … the condom [is] something that I don’t agree on.”[51]

Some women developed strategies to convince their partners, “You should speak to him in a nice way instead of just saying “use”! You should convince him in the way you convince a small child… explaining to him… And remind him of what fate would befall our family and children if we get the virus.”[51] Achan’s study on young mothers in Ghana indicated that some women with more education who were more financially stable left or threatened to leave their partners as a strategy to push for greater condom use.[75]

Most studies addressed, either directly or indirectly, that while HIV prevention may be important, other priorities, like preserving a relationship, earning money, being a good parent, or conforming, may be more valued at certain times in a person’s life.[50–53, 56, 58, 60, 62, 64, 65, 67, 68, 70, 71, 73, 74, 76, 78] For example, women may want to use condoms, but in trying to negotiate their use, risk upsetting or losing their partner. This loss of emotional or material support may be perceived as more consequential thana potential infection. In research on student nurses in Zimbabwe, one woman clearly articulated the difficult balance between wanting to be safe *and* wanting to be loved: “I asked my boyfriend to go for an HIV test together with me, but he is reluctant. He keeps on postponing, which is a sign that he does not want. Now he appears to have lost interest in me because I have said no to sex before being tested. Now, if all men are like that, what do I do?”[74] In some instances the certainty of a break up, argument or violence may be more immediate and personally significant than the comparatively abstract prospect of HIV infection resulting from inconsistent intervention use.[74] These risks were most commonly reported in the literature on condoms, which require consistent use and consent from the male partner to be effective. These concerns were much less common in studies on VMMC, a one-time procedure.[48, 77]

#### Positive symbolism makes intervention use desirable

Some prevention interventions had positive symbolic meaning in certain contexts,[54, 59, 73, 74, 77], making them more desirable to use. Moreover, having personal goals, like wanting a serious relationship or children with one’s partner, encouraged people to examine their HIV risk and motivated intervention use.

VMMC was viewed by some men as a “responsible choice”, a “symbol of commitment” and reflected maturity and commitment to future partners, making them more desirable as partners.[77] Moreover, its value as an HIV prevention tool was secondary to its increasing social value including perceived hygiene, cleanliness, and sex appeal.[63] One participant in a study in northern Malawi said, “I did not want to be a subject of discussion, so I decided to yank it out [get circumcised]. Now I am happy that I don’t really have to worry about embarrassment [of being uncircumcised].”[63]

A supportive relationship motivated some couples to seek HIV testing, despite high potential social, personal, and material costs of testing positive: “I went for HIV and AIDS testing and counselling when my husband said we should. But I was afraid of the possible consequences as you may end up pointing fingers about who is responsible. But my husband was supportive and promised me there would be no blaming game” explained a female student nurse.[74] When discussing whether or not to go for HIV testing with married couples, Chirwa et al interviewed a couple in Malawi who explained that, “both of us initiated this. It was as if we were thinking along the same lines…both of us have had the test four times. Now we just encourage each other because we are not infected by HIV.”[60] Being tested and receiving a negative result fortified their trust and love.

In the studies included in this review, those published most recently discussed condoms as a valuable resource for preserving their health and the health of their partners, despite some of the challenges reported.[54, 57] A man in Stern et al’s study explained, “I do not want to teach myself to get used to not using a condom so that I can put other people’s lives in danger.”[54] Another man from the same study said, “I was not really worried about me. I was worried about other people, of putting other people’s lives in danger. The most depressing thing is to think that other people can die because of you. Even today that is the reason that makes me use a condom—as a responsible person.”[54]

The educational component of VMMC also appears to influence health preserving behaviours, especially around condom use: “I can tell her that despite being circumcised, we must continue using a condom because MC [male circumcision] is not 100 percent. It only prevents 60 percent. Therefore, for us to protect better, we must use a condom… I can tell her that she must also be faithful to me, because if she has an affair outside marriage, she can still infect me even though I’m circumcised.”[57] The study did not test the sustainability of these changes in attitude or behaviour.

### Additional considerations for sex workers

In addition to the challenges discussed above, sex workers face further risks regarding HIV prevention, as discussed below.

#### Poverty, risk perception and intervention use

Sex workers repeated the role of poverty in multiple aspects of sex work.[58, 65, 67, 68, 73, 76] Many felt that sex work was their only option to provide for themselves or their families and emphasised its role in pushing them towards more unsafe but financially rewarding sexual practices.[52, 58, 65, 67–69, 73] One woman reported, “I need to hurry and get as much money before the sickness comes. I have to have the money… it’s very hard. What will happen to my children when I die? If a man will pay big money for sex without condom I will do [it].”[69] The risk and sense of inevitability of infection expressed by this respondent is clear, as is her need to prioritise her children’s well-being over her own. Her need for money outweighs her ability to refuse sex.

Some sex workers however, reported consistent condom use. In Ohnishi and Notiҫo’s study on a peer-led intervention in Mozambique, all sex workers reported consistent condom use.[66] In two other studies [65, 73] some respondents reported refusing unprotected sex regardless of the pay: “I always force my clients to use a condom. I have never accepted having sex without a condom. Even if a client is a regular, we have to use a condom.”[65] For these women, risk of HIV is omnipresent and intervention use is non-negotiable.

#### Relationships between sex workers and clients

The relationships that sex workers had with their clients varied widely. One study from Madagascar examined in detail the fluidity of these relationships.[76] Stoebenau et al found that commercial relationships often transitioned quickly to romantic relationships. During that transition, pay for sex decreases while dependence on the other person for assistance increases. This fluidity can trap women in cycles of falling in love with clients thus reducing their power to negotiate intervention use. While clients who become partners may offer some material support, it is often insufficient for her survival, and they may become jealous of her relations with other clients, which he interprets as infidelity.[76] In Nigeria, Izugbara found that some sex workers constructed condomless sex as a sign of her client’s responsibility and trust: “It means you trust your partner, you are confident he will not deliberately want to harm you”[68] also showing the fluidity between being a client and a partner.

Sex workers reported common manipulations by clients to have unprotected sex. One woman in Nigeria reported that her clients say, “‘Don’t you love me? I love you and know you’re clean. I believe you don’t have anything in your body. I trust you … don’t you love me … you don’t love me? I’m not your client now I’m your boyfriend … how can we get married if you continue like this?’ Sometime[s] we use condom[s], but most time[s] we don’t. I love him and I don’t want this work all my life.”[69] Paired with the desire to leave sex work and enter into a loving partnership, condom use falls in people’s hierarchy of concerns.

## Discussion

This review found evidence that examining risk perception of HIV in isolation is insufficient for understanding intervention use. Interventions themselves are replete with risks that may threaten relationship stability, economic security, and may be incompatible with the desire for a committed or loving relationship. To understand intervention use, risk should be conceptualized within the wider context in which decisions are made. Interventions themselves carry economic, social, and emotional risks associated with them, as outlined in the introduction. When HIV infection feels inevitable, using prevention seems inconsequential.

Health decisions involve consideration of how likely different possible outcomes are, and their various short- and long-term effects. The short-term effects may be felt more immediately and their likelihood may be easier to judge. Potential long-term consequences may involve more uncertainty and the span of time may make future risk more difficult to grasp. For example, avoiding conflict with a spouse may be more important than demanding condom use to prevent a potential HIV infection.

Moreover, the risks emanating from one intervention may not transfer to others. The majority of the evidence discusses condom use but their unique attributes, including the need for male approval and consistent use may not translate to interventions like PrEP, which can be used covertly and during seasons of risk.

In Corneli’s study on HIV worry among women who seroconverted while participating in the FEM-PrEP study in Kenya and South Africa, 52% of those who had contracted HIV had reported that there was no chance they would become infected in the next four weeks.[50] The study authors postulate four risk rationalisations, which made participants feel invulnerable: protective behaviour (engaging in at least one HIV prevention practice), protective reasoning (acknowledging risk but rationalising that there was no need to worry), recognised vulnerability, and those who did not rationalise their risk or take any actions to prevent infection. Therefore, risk perception and use of prevention interventions varied widely-some respondents were able to engage in more protective behaviour than others.[50]

Research on the acceptability of a new intervention often hinges on product use and its attributes. While these factors impact peoples’ willingness to use them, there is also a need to understand how people understand the meaning of the intervention. Condoms are symbolic of infidelity, mistrust, and therefore only relevant in short-term relationships. VMMC appears to be connected to a much more positive symbolic meaning of responsibility, cleanliness, and increased sexual pleasure. Emerging interventions, including PrEP, microbicides, intravaginal rings, and an HIV vaccine, have a unique opportunity to ‘brand’ their meaning with associations compatible with love, commitment, fidelity, responsibility, and sexual pleasure.[79] However, interventions marketed as female-controlled and empowering may result in male resistance. Preventing HIV was important to respondents in all studies but was not their most pressing concern. By making HIV prevention compatible with, and integral to, their larger personal concerns, HIV prevention may become more relevant.

There are limitations to this review that should be considered. It was not feasible to contextualise all of the findings from such a diverse area as sub-Saharan Africa in terms of language, culture, health systems, and HIV epidemic, but we aimed to identify key themes that could be useful to researchers, policy-makers, and clinicians. We did not consider grey-literature which may have additional unique and valuable insights.

Despite these limitations, there is considerable evidence that risks extend beyond disease transmission and enter into deeply personal realms of trust, love, economic security, and values. As new HIV prevention interventions emerge, there are opportunities to endow them with symbolism connected to trust, love, and feelings of personal autonomy. Healthcare providers and clinical trialists need to be mindful that interventions are appropriate not only based on clinical but also situational and personal indicators.

### Conclusion

Decision-making around HIV prevention interventions is influenced by multiple factors beyond specific concerns regarding disease prevention. The use of prevention interventions carries personal and symbolic risks, which must be considered. Emerging technologies have a unique opportunity to ‘brand’ themselves with positive social connotations, facilitating their use.

Interventions are not merely physical commodities; they are steeped in symbolic meaning.[80] For example, microbicides have been found to be imbued with meanings of empowerment and hope.[79] Emerging interventions, whose symbolic meanings are being constructed anew, may be uniquely positioned to infuse their ‘brand’ with associations compatible with love, commitment, responsibility, and sexual pleasure, rather than those associated with disease, danger, and distrust. If interventions have positive symbolic meaning and are understood to have fewer risks associated with them, uptake and adherence may improve.

## Supporting information

S1 Table Search strategy. (DOCX)

S2 Table PRISMA checklist. (DOCX)
