# Supplementary material for: Risk perception and the influence on uptake and use of biomedical prevention interventions for HIV in sub-Saharan Africa: A systematic literature review
Source: PLoS One. 2018 Jun 14;13(6):e0198680. doi: 10.1371/journal.pone.0198680 (PMC6002067; doi:10.1371/journal.pone.0198680)
Supplement: S1 Table — (DOCX) [file pone.0198680.s001.docx]

# **Supplement 1: Search Strategy**

| **Concepts** | **Free Text**  (used across all databases) | **Databases and Mesh Terms** | | | | **EBSCOHost**  (free-text only) | |
| --- | --- | --- | --- | --- | --- | --- | --- |
|  | (.ti, ab for HIV, risk, uptake and qualitative; .mp for Sub-Saharan Africa) | **Medline** (adj#) | **Embase** (adj#) | **PsychInfo** (adj#) | **Global Health** (adj#) | **AfricaWide Info** (N#) | **CINAHL** (N#) |
| **HIV PREVENTION** | ((hiv or hiv-1 or hiv2* or hiv1 or hiv2 or hiv infect* or human immunodeficiency virus or human immune deficiency virus or human immuno-deficiency virus or human immune-deficiency virus or acquired immunodeficiency syndromes or acquired immune deficiency syndrome or acquire immuno-deficiency syndrome or acquired immune-deficiency syndrome) adj3 (prevent* or avert* or reduc* or suppress* or limit*))  (((human immun* and deficiency virus) or (acquired immun* and deficiency syndrome) or HIV#AIDS) adj3 (prevent* or avert* or reduc* or suppress* or limit*))  (pre?exposure prophylaxis or PrEP or post?exposure prophylaxis or PEP or treatment as prevention or TASP or condom? or microbicide? or circumcision or (ST* treat) or (treat* adj3 ST*)) | HIV Infections/ HIV/ Condoms/  Primary Prevention/ | Human immunodeficiency virus infection/ Human immunodeficiency virus/ preventive health service/ Prevention/ Prophylaxis/ post exposure prophylaxis/  condom/  “condom use”/  circumcision/ | HIV/ AIDS Prevention/ condoms/ | human immunodeficiency viruses/ HIV infections/ disease prevention/ prophylaxis/ condoms/ needle exchange schemes/ |  | (MH "HIV Infections")  (MH "human immunodeficiency virus")  (MH "Condoms")  (MH "Circumcision") |
| **RISK** | (risk or risk* or risk perception* or concern* or ambigu* or uncertain* or danger or chance or misfortune or trust or distrust or mistrust or hope or guess or speculat* or vulnerabil* or invulnerabil* or secur* or insecure* or connect* or power or control or confidenc*)  perce* adj3 risk* | Risk-Taking/ Risk Assessment/ Perception/ Uncertainty/ Hope/ Trust/ | danger, risk, safety and related phenomena/ risk assessment/ risk management/ trust/ hope/ | Risk Perception/ Risk Assessment/ Sexual Risk Taking/ Uncertainty/ Trust (Social Behavior)/ Interpersonal Control/ | Risk/ Risk Analysis/ Risk Assessment/ Risk Behavior/ Uncertainty/ Perception/ |  | (MH "Attitude to Risk")  (MH "Risk Taking Behavior")  (MH "Risk Assessment")  (MH "Trust")  (MH "Control (Psychology)")  (MH "Hope")  (MH "Uncertainty") |
| **UPTAKE** | (uptake or adopt or take-up or use* or usage or effectiveness or coverage or access or decision-making or adherence or drop-out* or dropout* or compliance or complie* or comply or encourage* or improve* or increas* or promot* or participat* or nonattend* or accept* or attend* or attitude* or utili?ation or refus* or reluctan* or determinant* or driver* or factor* or motivat* or predict*) | Attitude to Health/ Patient Compliance/ Decision Making/ Patient Acceptance of Health Care/ | patient compliance/ attitude to health/ decision making/ | Treatment/ Treatment Barriers/ Treatment Compliance/ Treatment Dropout/ Treatment Refusal/ Health Knowledge/ Health Attitudes/ Health Behavior/ Decision making/ Client attitudes/ | patient compliance/ decision making/ |  | (MH "Attitude to Health")  (MH "Attitude to Illness")  (MH "Patient Compliance")  (MH "Decision Making, Patient")  (MH "Treatment Refusal")  (MH "Health Knowledge") |
| **QUALITATIVE RESEARCH** | (semi-structured or semistructured or unstructured or open-ended or openended or informal or formal or in-depth or indepth or face-to-face or structured or guide* or interview* or discussion* or ((field adj2 stud*) or research) or focus group* or qualitative or ethnograph* or field-work or fieldwork or key-informant or mixed-method* or finding*) | Qualitative research/  Interviews as topic/ personal narratives/ narration/ focus groups/ | qualitative research/ interview/ | Qualitative research/ Interviews/ Group Discussion/ Narratives/ | Qualitative analysis/ Interviews/ |  | (MH "Qualitative Studies")  (MH "Interviews")  (MH "Semi-Structured Interview")  (MH "Unstructured Interview")  (MH "Structured Interview")  (MH "Focus Groups") |
| **SUB-SAHARAN AFRICA** | (Africa adj2 south*) or (subsahara* adj2 africa) or (Africa adj2 south adj2 sahara*) or Angola or (sao tome adj2 principe) or (Benin or Dahomey) or (Botswana or Bechuanaland or Kalahari) or (Burkina Faso or Burkina Fasso or Upper Volta) or Burundi or Cameroon or Cape Verde or (Central African Republic or Ubangi-Shari) or Chad or (Comoros or Comoro Islands or Mayotte or Iles Comores) or (congo not ((democratic republic adj3 congo) or congo red or crimean-congo)) or (Cote d'Ivoire or Ivory Coast) or ((democratic republic adj2 congo) or belgian congo or zaire) or Eritrea or Ethiopia or (Gabon or Gabonese Republic) or Gambia or (Ghana or Gold Coast) or (Guinea not (New Guinea or Guinea Pig* or Guinea Fowl)) or (Guinea-Bissau or Portuguese Guinea) or Kenya or (Lesotho or Basutoland) or Liberia or (Madagascar or Malagasy Republic) or (Malawi or Nyasaland) or Mali or Mauritania or (Mauritius or Agalega Islands) or (Mozambique or Portuguese East Africa) or Namibia or (Niger not (Aspergillus or Peptococcus or Schizothorax or Cruciferae or Gobius or Lasius or Agelastes or Melanosuchus or radish or Parastromateus or Orius or Apergillus or Parastromateus or Stomoxys)) or Nigeria or (Rwanda or Ruanda) or Senegal or Seychelles or Sierra Leone or Somalia or South Africa or South Sudan or Sudan or Swaziland or (Tanzania or Zanzibar) or (Togo or Togolese Republic) or Uganda or (Zambia or Northern Rhodesia) or (Zimbabwe or Rhodesia) | Africa South of the Sahara/ or Angola/ or Atlantic Islands/ or Benin/ or Botswana/ or Burkina Faso/ or Burundi/ or Cameroon/ or Cape Verde/ or Central African Republic/ or Chad/ or Comoros/ or Congo/ or Cote d'Ivoire/ or "Democratic Republic of the Congo"/ or Eritrea/ or Ethiopia/ or Gabon/ or Gambia/ or Ghana/ or Guinea/ or Guinea-Bissau/ or Kenya/ or Lesotho/ or Liberia/ or Madagascar/ or Malawi/ or Mali/ or Mauritania/ or Mauritius/ or Mozambique/ or Namibia/ or Niger/ or Nigeria/ or Rwanda/ or Senegal/ or Seychelles/ or Sierra Leone/ or Somalia/ or South Africa/ or South Sudan/ or Sudan/ or Swaziland/ or Tanzania/ or Togo/ or Uganda/ or Zambia/ or Zimbabwe/ | Africa South of the Sahara/ or Angola/ or Atlantic Islands/ or Benin/ or Botswana/ or Burkina Faso/ or Burundi/ or Cameroon/ or Cape Verde/ or Central African Republic/ or Chad/ or Comoros/ or Congo/ or Cote d'Ivoire/ or "Democratic Republic of the Congo"/ or Eritrea/ or Ethiopia/ or Gabon/ or Gambia/ or Ghana/ or Guinea/ or Guinea-Bissau/ or Kenya/ or Lesotho/ or Liberia/ or Madagascar/ or Malawi/ or Mali/ or Mauritania/ or Mauritius/ or Mozambique/ or Namibia/ or Niger/ or Nigeria/ or Rwanda/ or Senegal/ or Seychelles/ or Sierra Leone/ or Somalia/ or South Africa/ or South Sudan/ or Sudan/ or Swaziland/ or Tanzania/ or Togo/ or Uganda/ or Zambia/ or Zimbabwe/ |  | Africa South of the Sahara/ or Angola/ or Atlantic Islands/ or Benin/ or Botswana/ or Burkina Faso/ or Burundi/ or Cameroon/ or Cape Verde/ or Central African Republic/ or Chad/ or Comoros/ or Congo/ or Cote d'Ivoire/ or "Democratic Republic of the Congo"/ or Eritrea/ or Ethiopia/ or Gabon/ or Gambia/ or Ghana/ or Guinea/ or Guinea-Bissau/ or Kenya/ or Lesotho/ or Liberia/ or Madagascar/ or Malawi/ or Mali/ or Mauritania/ or Mauritius/ or Mozambique/ or Namibia/ or Niger/ or Nigeria/ or Rwanda/ or Senegal/ or Seychelles/ or Sierra Leone/ or Somalia/ or South Africa/ or South Sudan/ or Sudan/ or Swaziland/ or Tanzania/ or Togo/ or Uganda/ or Zambia/ or Zimbabwe/ |  | (MH "Africa South of the Sahara") or (MH “Angola”) or (MH “Atlantic Islands”) or (MH “Benin”) or (MH “Botswana”) or (MH “Burkina Faso”) or (MH “Burundi”) or (MH “Cameroon”) or (MH Cape Verde”) or (MH “Central African Republic”) or (MH “Chad”) or (MH “Comoros”) or (MH “Congo”) or (MH “Cote d'Ivoire”) or (MH "Democratic Republic of the Congo") or (MH “Eritrea”) or (MH “Ethiopia”) or (MH “Gabon”) or (MH” Gambia”) or (MH “Ghana”) or (MH “Guinea”) or (MH “Guinea-Bissau”) or (MH “Kenya”) or (MH “Lesotho”) or (MH “Liberia”) or (MH “Madagascar”) or (MH “Malawi”) or (MH “Mali”) or (MH “Mauritania”) or (MH “Mauritius”) or (MH “Mozambique”) or (MH “Namibia”) or (MH “Niger”) or (MH “Nigeria”) or (MH “Rwanda”) or (MH “Senegal”) or (MH “Seychelles”) or (MH “Sierra Leone”) or (MH “Somalia”) or (MH “South Africa”) or (MH “South Sudan”) or (MH “Sudan”) or (MH “Swaziland”) or (MH “Tanzania”) or (MH “Togo”) or (MH “Uganda”) or (MH “Zambia”) or (MH “Zimbabwe”) |
